# Development of a novel autophagy-related gene model for gastric cancer prognostic prediction

**DOI:** 10.3389/fonc.2022.1006278

**Published:** 2022-10-07

**Authors:** Haifeng Xu, Bing Xu, Jiayu Hu, Jun Xia, Le Tong, Ping Zhang, Lei Yang, Lusheng Tang, Sufeng Chen, Jing Du, Ying Wang, Yanchun Li

**Affiliations:** ^1^ Laboratory Medicine Center, Department of Clinical Laboratory, Zhejiang Provincial People’s Hospital (Affiliated People’s Hospital, Hangzhou Medical College), Hangzhou, China; ^2^ School of Laboratory Medicine and Life Science, Wenzhou Medical University, Wenzhou, China; ^3^ Department of Clinical Laboratory, Hangzhou Women’s Hospital, Hangzhou, China; ^4^ College of Medical, Veterinary and Life Sciences, University of Glasgow, Glasgow, United Kingdom; ^5^ Department of Central Laboratory, Affiliated Hangzhou first people’s Hospital, Zhejiang University School of Medicine, Hangzhou, China

**Keywords:** gastric cancer, autophagy-related genes, prognostic model, tumor immunity, drug resistance

## Abstract

Gastric cancer (GC) is a major global health issue and one of the leading causes of tumor-associated mortality worldwide. Autophagy is thought to play a critical role in the development and progression of GC, and this process is controlled by a set of conserved regulators termed autophagy-related genes (ATGs). However, the complex contribution of autophagy to cancers is not completely understood. Accordingly, we aimed to develop a prognostic model based on the specific role of ATGs in GC to improve the prediction of GC outcomes. First, we screened 148 differentially expressed ATGs between GC and normal tissues in The Cancer Genome Atlas (TCGA) cohort. Consensus clustering in these ATGs was performed, and based on that, 343 patients were grouped into two clusters. According to Kaplan–Meier survival analysis, cluster C2 had a worse prognosis than cluster C1. Then, a disease risk model incorporating nine differentially expressed ATGs was constructed based on the least absolute shrinkage and selection operator (LASSO) regression analysis, and the ability of this model to stratify patients into high- and low-risk groups was verified. The predictive value of the model was confirmed using both training and validation cohorts. In addition, the results of functional enrichment analysis suggested that GC risk is correlated with immune status. Moreover, autophagy inhibition increased sensitivity to cisplatin and exacerbated reactive oxygen species accumulation in GC cell lines. Collectively, the results indicated that this novel constructed risk model is an effective and reliable tool for predicting GC outcomes and could help with individual treatment through ATG targeting.

## Introduction

With more than 1 million new cases diagnosed annually and approximately 783,000 deaths reported in 2018, gastric cancer (GC) is considered the fifth most common malignancy and the third leading cause of cancer-related mortality globally (1, 2). Currently, the predominant treatment strategies for GC are surgery, chemoradiotherapy, molecular-targeted therapies, and immunotherapy (3, 4). Despite great improvements in treatments, the current therapeutic strategy remains unsatisfactory (5). Owing to the lack of specific diagnostic biomarkers for GC, most initially asymptomatic patients are diagnosed at the late stages of the disease and thus have a poor prognosis (6). To date, prognostication in oncology has been largely based on tumor-node-metastasis (TNM) staging guidelines (7). Nevertheless, the high heterogeneity of GC could lead to distinct clinical outcomes for patients even if they have similar clinicopathological characteristics, suggesting that the current TNM staging guidelines are not satisfactory prognostic tools for disease risk stratification (8, 9). Therefore, to ameliorate the typically poor outcomes of patients with GC, new prognostic models that utilize novel biomarkers to stratify GC patients are urgently needed.

Great progress has been made in recent years in terms of understanding tumor pathogenesis and progression, particularly the effects of autophagy in cancers (10). Autophagy was originally defined by de Duve in 1963 as the programmed self-digestion of the cell (11). Autophagy, which serves as a primary mechanism for the maintenance of cellular homeostasis, can be viewed as a critical lysosome-dependent catabolic process in eukaryotic cells and is responsible for the turnover of organelles and proteins through lysosomal degradation (12, 13). There have been several reports of the correlation between autophagy and poor prognosis, increased metastasis, and chemoresistance (14, 15). Excessive autophagy can trigger type II programmed cell death, which differs from other programmed cell death processes, such as apoptosis and ferroptosis (16, 17). Accumulating evidence indicates that abnormal autophagy is involved in multiple types of cancer, including esophageal, gastric, and breast cancer (18–20). In contrast to other cancers, GC is characterized by an elevated level of autophagy, which could account for the associated high rates of cell death (21, 22). We thus reasoned that the induction of autophagic cell death in cancer cells holds promise as a useful supplement to current treatments for GC and that the identification of molecular-defined targets might help improve the prognosis of patients with GC.

In the past decade, autophagy has attracted much interest as a research topic, leading to the discovery of a large number of autophagy-related genes (ATGs) that control this process (23). Previous studies have shown that the expression levels of several ATGs (e.g., *Beclin1*, *LC3*, and *P62*/*SQSTM1*) significantly affect the prognosis of GC (24–26). Therefore, we reasonably inferred that ATGs might be promising tools for GC prognostication. Given the heterogeneity of disease severity and outcomes of GC, a multigene-based prognostic model is expected to be superior to previous single-gene biomarkers in predicting GC outcomes. Therefore, we established a risk model based on nine ATGs, which might provide more options for predicting the prognosis of GC and offer clinicians more promising therapeutic targets.

We first clustered patients with GC after analyzing the expression levels of ATGs from TCGA database. The least absolute shrinkage and selection operator (LASSO) regression analysis was then performed to screen ATGs that were markedly survival-related, and a nine-gene prognostic model was confirmed to have the ability to stratify patients into different risk groups. The specificity and sensitivity of the model were verified using a Gene Expression Omnibus (GEO) cohort, and the performance of the new model was assessed using a receiver operating characteristic (ROC) curve and Kaplan–Meier (K–M) survival analysis. Finally, we compared immune infiltration activity between the subgroups. Moreover, we found that autophagy inhibition increased sensitivity to cisplatin (DDP) and exacerbated reactive oxygen species (ROS) accumulation in GC cell lines. Collectively, our results showed that the established prognostic model provides an effective and trustworthy strategy for outcome prediction in GC and might help with individual treatments targeting ATGs.

## Methods

### Datasets

The GC samples were obtained from TCGA Data Portal (https://portal.gdc.cancer.gov/repository). According to the 14^th^ and 15^th^ digits of the sample barcode, tumor tissues ranged from 01-09 and normal tissues from 10-19. Finally, the transcriptome profiles of 343 tumor samples and 30 normal samples were extracted from TCGA database. The RNA-seq data and the corresponding clinical information were acquired for further research. For validation, the GSE84437 dataset and GSE26942 dataset were retrieved from the Gene Expression Omnibus (GEO dataset https://www.ncbi.nlm.nih.gov/geo/). The FPKM values of TCGA-STAD were transformed into transcripts per kilobase million.

### Defining differentially expressed ATGs

232 ATGs were collected from the Human Autophagy Database (http://www.autophagy.lu/index.html) which were listed in **Table S1**. Gene expression values were log2 transformed, then the differentially expressed genes (DEGs) between GC and adjacent normal tissues were identified by the “limma” package with |logFC| ≥1 and adjusted *p*< 0.05 (**Table S2**). For visualizing the differences in gene expression, a heatmap was performed with the “pheatmap” R package. Utilizing the R program “igraph” and “reshape2”, the correlation of DEGs was examined, and the results were presented in the expressional correlation networks with the cutoff score of 0.4. Protein-protein interaction (PPI) network for the DEGs with an interaction score of 0.9 was built by using the Search Tool for the Retrieval of Interacting Genes (STRING v11.5, https://cn.string-db.org/).

### Consensus clustering analysis of ATGs

In TCGA cohort, consensus clustering was employed to cluster GC patients into two clusters by the R packages “limma” and “ConsensusClusterPlus” based on the expression of ATGs. The optimal cluster number k = 2 was selected according to the cumulative distribution function. Variances in clinical characteristics of the two clusters were explored by the chi-squared test and “survival” package. The heatmap and K–M curves visualized the results *via* R packages “pheatmap”, “survival”, and “survminer”.

### Establishing and validating the prediction model

The prognostic and predictive value of DEGs were examined by the univariate Cox analysis in TCGA cohort. Then, 17 ATGs were identified as significantly survival-related genes with *p*<0.001. To prevent overfitting, the LASSO regression analysis was performed with the R package “glmnet”. Ultimately, nine candidate genes with their corresponding coefficients were kept. The penalization parameters (λ) were assessed by tenfold cross-validation based on minimum criteria. We established the final model according to the optimal λ which achieved the highest mean cross-validation areas under the curves (AUC). Then we assigned every patient a risk score with the formula below: risk score = $\sum_{k=1}^{n}\exp k*l\;$ (where n, exp *k*, and *l* represent the gene number, gene expression level, and the coefficient of gene *k*, respectively). The median cut-off of the risk score was considered to allocate patients into two different risk subgroups. Principal component analysis (PCA) and t-Distributed Stochastic Neighbor Embedding (t-SNE) analysis (R package “Rtsne” and “ggplot2”) visualized the distribution of two subgroups in terms of gene expression level. Besides, the K–M curves (“survminer” R package) and the time-dependent ROC curves (“timeROC” packages) were plotted to appraise the clinical value. To further validate the prognostic model, we carried out the same formula of the risk score in the GEO database (GSE84437) and found that patients were also divided into low- or high-risk subgroups. The model’s performance was examined in the validation cohort as well.

### Analysis of the expression levels of modeling genes

Protein expression levels of modeling genes were analyzed from the Human Protein Atlas (HPA, https://www.proteinatlas.org), which involves immunohistochemistry data. Immunohistochemical images of the proteins between normal and GC tissues were downloaded for omparisons. The microarray data of the GSE26942 dataset was downloaded from the GEO database (http://www.ncbi.nih.gov/geo) for additional validation at the transcriptional level.

### Evaluation of independent prognostic factors in GC patients

To identify risk factors associated with survival, univariate and multivariate Cox regression analyses were performed with R package "survival" in TCGA and GEO cohorts. The differences in clinical traits between the risk groups were analyzed by employing the chi-squared test and the result was presented by a heatmap.

### Functional enrichment of DEGs between distinct risk groups

In the training and testing cohorts, the DEGs filtered across the high- and low-risk groups with significance criteria (*p*<0.05) were further conducted with Gene ontology (GO) annotation and Kyoto Encylopedia of Genes and Genomes (KEGG) analysis by using the “clusterProfiler” package. The single-sample gene set enrichment analysis (ssGSEA) was adopted to assess immune-related pathway activity and infiltrating immune cell scores by applying the “GSVA” package. Furthermore, the “limma” and “pheatmap” R packages were utilized for immune deconvolution analysis between the risk groups in TCGA cohort through different algorithms, such as TIMER, CIBERSORT, CIBERSORT-Absolute, quanTIseq, MCPcounter, xCell, and EPIC. A heatmap was drawn to exhibit the combined results.

### Construction of nomogram in TCGA cohort

Based on the results of multivariate Cox analysis, we developed a nomogram by applying the R package “survival” and “regplot”, and evaluated the performance of the nomogram by the ROC curves. In the end, we performed the decision curve analysis (DCA) through the R package “survival” and “ggDCA” to compare the predictive power of the nomogram model with all the clinical features.

### Cell culture

The human gastric cancer cell line AGS was purchased from the Cell Bank of the Chinese Academy of Sciences (Shanghai, China). Cells were cultured in Dulbecco’s modified Eagle’s medium (DMEM, Hyclone, United States), containing 10% fetal bovine serum (FBS, Gibco, United States), 100 U/mL penicillin, and 100 µg/mL streptomycin (Beyotime, China). Cells were incubated at 37°C under a humidified atmosphere with 5% CO_2_.

### Reagents and antibodies

The antibody to β-Actin (Abcam, ab179467) was purchased from Abcam (Cambridge, MA). The antibody to LC3 (Sigma, L7543) was purchased from Sigma-Aldrich (St. Louis, USA). The corresponding HRP-conjugated secondary antibody was purchased from Beyotime (Shanghai, China). DDP and 2′,7′-dichlorofluore scin diacetate (DCF-DA) were obtained from Sigma-Aldrich (St. Louis, USA). Bafilomycin A1 (BafA1), 3-Methyladenine (3MA), and Chloroquine (CQ) were purchased from Medchem Express (MCE, United States). Hoechst 33342 was purchased from Solarbio (Beijing, China). Cell Count Kit-8 (CCK-8) Assay Kit was obtained from Meilunbio (Dalian, China).

### Cell viability assay

Cell viability was detected by CCK-8 kit assay. AGS cells (1 × 10^4^ per well) were seeded in 96-well plates (NEST Biotechnology) and cultured overnight until completely adherent. In the drug combination experiments, the cells were pretreated with autophagy inhibitors including BafA1(10µM), 3MA(3mM), and CQ (20µM) for 4 hours. Then DDP (40µM) was co-treated with them for 24 hours in the incubator. Subsequently, the CCK-8 solution (10µL) was added to each well for another 1.5 hours at 37°C. The absorbance of corresponding wells was measured at 450 nm on the microplate reader (Thermo, United States).

### ROS production measurement

The intracellular ROS level was detected using DCF-DA as previously described (27). Briefly, AGS cells were placed in a chamber confocal dish and cultured overnight. After drug treatments, AGS cells were washed with PBS. Then DCF-DA (4 μM) was co-staining with Hoechst 33342 for 30 min at 37°C away from light. Cells were finally washed with PBS again and representative images were captured under confocal microscope.

### Western blot

Following drug treatments, cells were lysed in RIPA buffer with protease and phosphorylation inhibitor cocktail (Thermo, Waltham, MA, United States) for 10 min. The protein concentration was measured by BCA Protein Assay Kit (Beyotime, China). The western blot assay was detected as previously described (27). The protein bands were visualized by an ECL-Plus chemiluminescence detection kit (Thermo Fisher Scientific), and images were visualized by Gel Imager (Bio-Rad) (28).

### Statistical analysis

R software version 4.1.2 and the R packages described previously were used to conduct the statistical analysis. Differences were judged statistically significant at *p*<0.05. Wilcoxon test was performed to assess the gene expression levels between the subgroups and the immune infiltration levels between the risk groups. The Pearson chi-square test was applied to compare categorical variables. K–M method and a two-sided log-rank test were applied for survival analysis.

## Results

### Discrepant expression of ATGs between normal and tumor tissues

**Figure 1:**
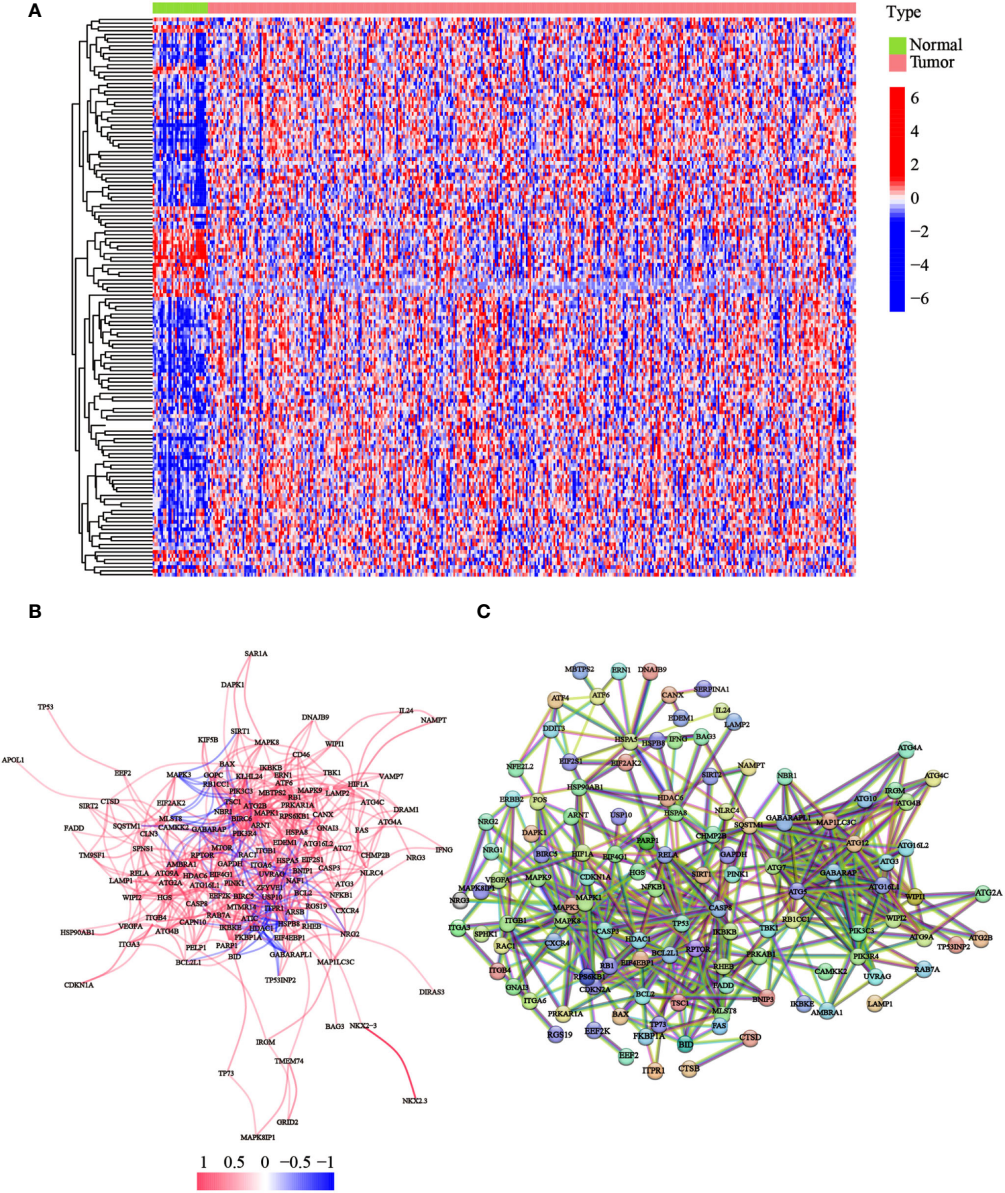
Expression of the ATGs and the interactions among these genes. **(A)** The heatmap displayed the variations in the gene expression levels of the differentially expressed ATGs between GC and normal tissues (red dot: high expression level; blue dot: low expression level). **(B)** Correlations of the ATGs were visualized through the expressional correlation networks (red line: positive correlations; blue line: negative correlations. The strength of the relevance was reflected in the depth of the colors). **(C)** PPI network exhibited potential interactions among these ATGs (interaction score = 0.9).

To detect the expression of ATGs, 373 total GC cases were acquired from TCGA database, of which, 30 cases were adjacent noncancerous samples. We initially analyzed the expression abundance of 232 ATGs that had been reported in previous studies. Of these, 148 genes were significantly differentially expressed between GC and normal tissues. The gene expression levels were visualized using a heatmap, which illustrated that most ATGs are highly expressed in GC tissues (**Figure 1A**). Expressional correlation networks (**Figure 1B**) and PPI analysis (**Figure 1C**) were performed to probe the potential pathways associated with the identified genes. After constructing the PPI network with the highest confidence interaction score level at 0.9, we identified 10 hub genes (*ARNT*, *ATF6*, *ATG10*, *ATG12*, *ATG16L1*, *ATG16L2*, *ATG3*, *ATG4B*, *ATG4B*, *ATG7*, and *BAG3*) that could be key genes in the autophagy process.

### Tumor cluster classification through consensus clustering of ATGs

**Figure 2:**
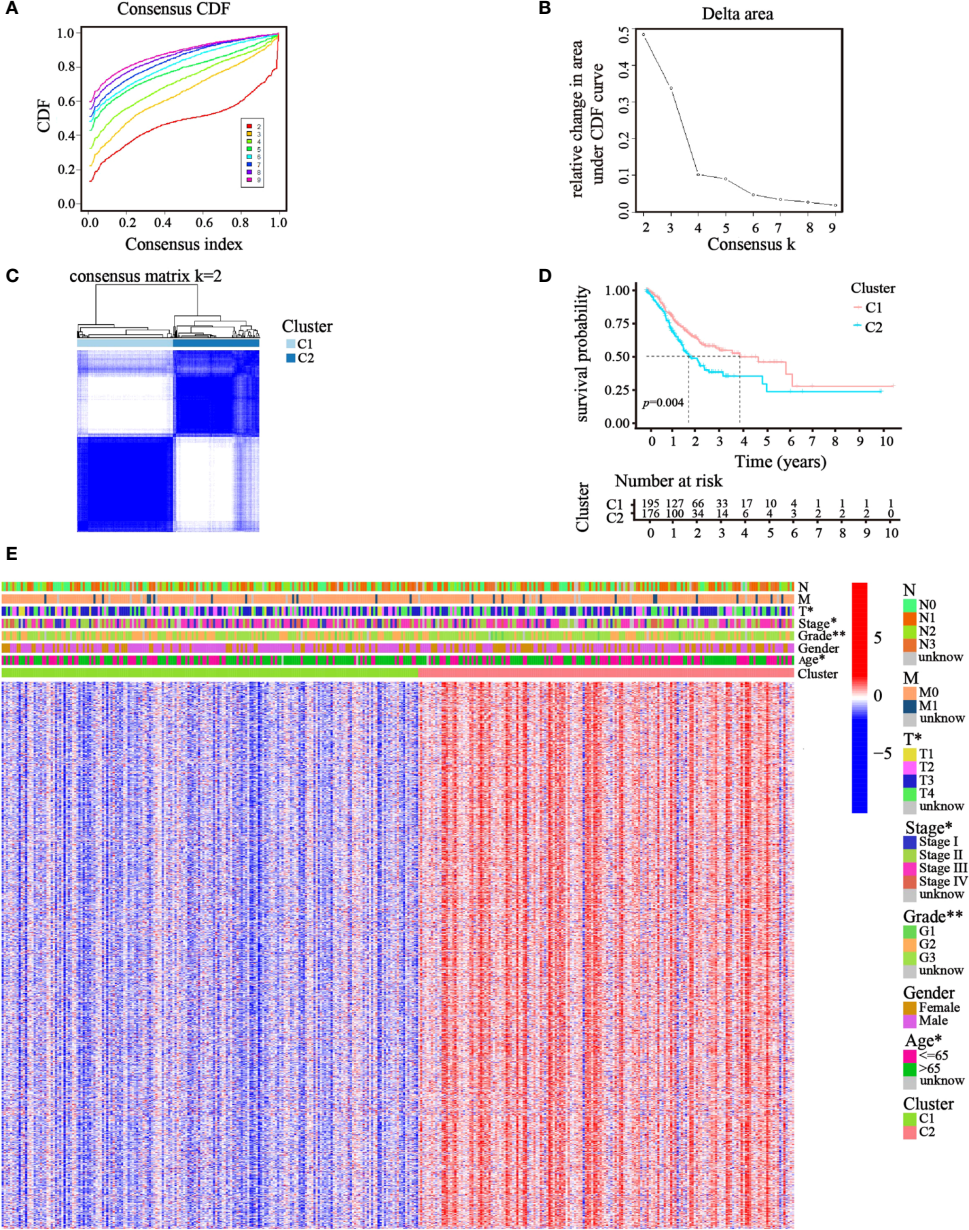
Clusters of GC patients based on ATGs in TCGA cohort. **(A)** CDF curves of different consensus k-values. The horizontal axis stood for the consensus index, and the vertical axis reflected CDF. **(B)** Delta area under the CDF curve. It was defined as the relative change in area under the CDF curve for each clustering number (k). **(C)** Heatmap of clustering at Consensus k = 2. **(D)** K–M curves showed survival differences between the clusters. **(E)** Differences in clinicopathologic characteristics and gene expression levels between the clusters were exhibited in the heatmap. (T, N, and M indicated the tumor-node-metastasis classification; *p< 0.05; **p< 0.01).

Focusing on the gene expression profiles of 148 ATGs, we identified different clusters through consensus clustering analysis based on 343 GC cases extracted from TCGA cohort. To obtain the clearest distinguished and the most robust clustering result, we clustered and ordered the patients using the clustering variable (k) from two to nine. The optimal k-value was determined by the Cumulative Distribution Function (CDF) and CDF Delta area curve (**Figures 2A, B**). Finally, an obvious trend of intergroup separation and intragroup aggregation emerged simultaneously when k = 2, which would represent the optimal selection for sorting (**Figure 2C**). Heatmaps of clustering results with other k-values are shown in the **Supplementary Materials** (**Figure S1**). A higher survival advantage was also observed for cluster 1 (**Figure 2D**). Subsequently, a heatmap was generated to further evaluate the clinicopathological features of the two clusters. Most genes showed a high expression level in cluster 2. There were also significant differences between the clusters in terms of age, tumor stage, tumor T stage, and tumor grade (*p*< 0.05; **Figure 2E**).

### Risk model based on prognosis-related ATGs

**Figure 3:**
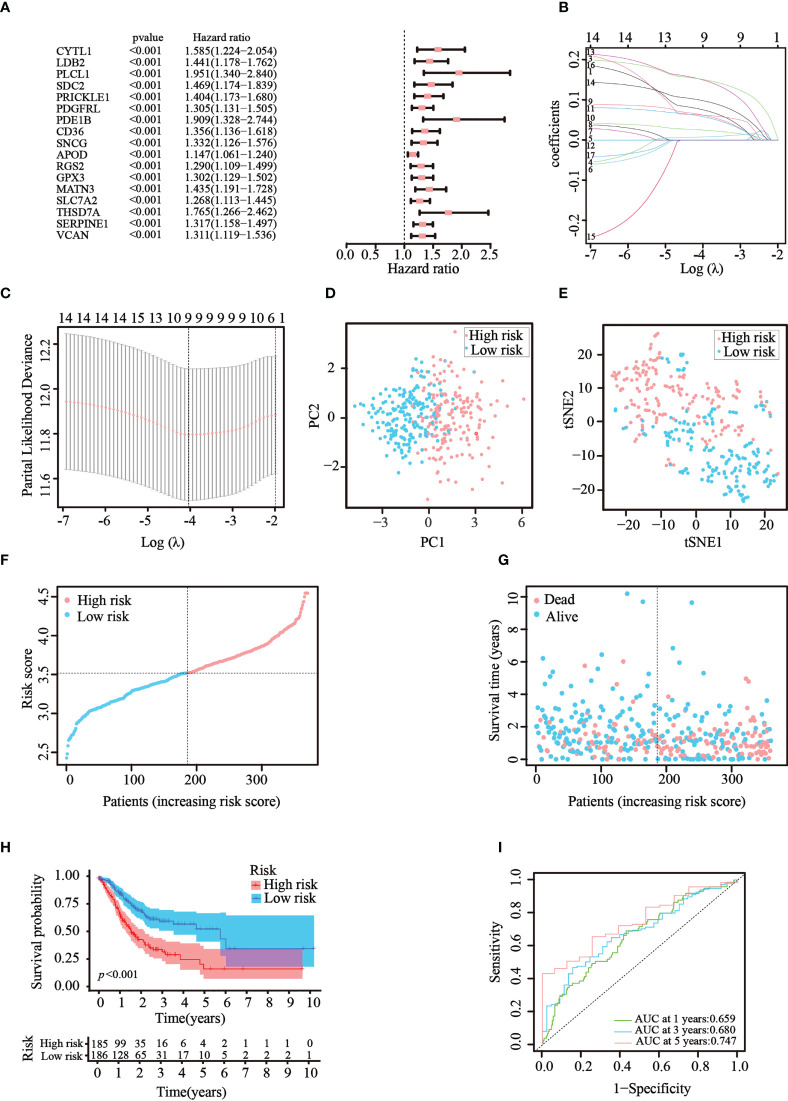
Construction of the prognostic risk model in TCGA cohort. **(A)** 17 survival-related genes were screened by univariate Cox regression analysis (*p*< 0.05). **(B, C)** By applying the tenfold cross-validation, optimal parameter λ was obtained in the LASSO regression. The final model was constructed with nine prognostic genes. **(D, E)** PCA and t-SNE analysis confirmed the good separation of the two risk categories. **(F, G)** Line and scatter charts revealed the distribution of each patient’s risk score and survival status. **(H)** K–M curves displayed the outcome of patients in the two risk subgroups. **(I)** A time-dependent ROC curve was drawn to assess the survival rates in 1, 3, and 5 years.

Then, univariate Cox regression analysis was used to screen the differentially expressed genes related to prognosis. We discovered that 17 genes were significantly correlated with the prognosis of GC patients and acted as risk factors (hazard ratio > 1; **Figure 3A**). LASSO regression was then implemented to narrow down the candidate genes and eliminate the risk of overfitting with the aforementioned genes. Ultimately, nine genes (*CYTL1*, *PLCL1*, *SNCG*, *APOD*, *RGS2*, *GPX3*, *MATN3*, *SLC7A2*, and *SERPINE1*) were retained to construct the prediction model following the optimum value (**Figures 3B, C**). The risk score was calculated using the following formula: risk score = (0.0763 × expression quantity of *CYTL1*) + (0.0625 × expression quantity of *PLCL1*) + (0.0630 × expression quantity of *SNCG*) + (0.0252 × expression quantity of *APOD*) + (0.0590 × expression quantity of *RGS2*) + (0.0077 × expression quantity of *GPX3*) + (0.1508 × expression quantity of *MATN3*) + (0.0989 × expression quantity of *SLC7A2*) + (0.1515 × expression quantity of *SERPINE1*). Employing the median cut-off value of the risk score, patients with GC were stratified into two risk subgroups in the training set. PCA and t-SNE analysis confirmed that patients with different risks were sorted well into different categories (**Figures 3D, E**). Additionally, the risk curve and scatter chart depicted the risk score distributions and survival statuses of all patients with GC. High-risk patients had a shorter survival time and higher fatality rate than low-risk individuals (**Figures 3F, G**). Consistently, K–M analysis illustrated that individuals in the high-risk group generally had worse survival rates than those in the low-risk group (*p* **<** 0.001, **Figure 3H**). To further assess the predictive efficacy of the prognostic model, time-dependent ROC analysis was structured with an AUC of 0.659 at 1 year, 0.680 at 3 years, and 0.747 at 5 years, indicating the good performance of our risk model in predicting overall survival (**Figure 3I**). Collectively, these analyses confirmed that the nine identified ATGs could compose a prognostic model for GC.

### Risk model validation using the GEO testing cohort

**Figure 4:**
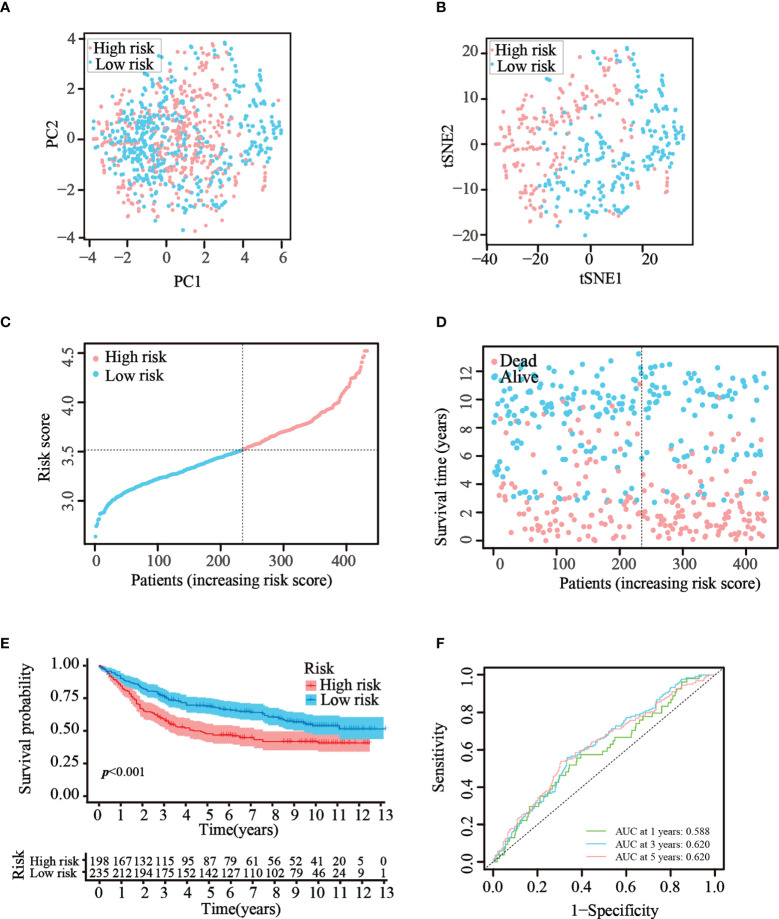
Validation of the prognostic risk model in the GEO dataset. **(A, B)** Scatter plot of PCA and t-SNE analysis in the GEO dataset. **(C, D)** Distribution of patients and their survival statuses in the GEO dataset. **(E)** K–M curves were drawn to compare the survival time of the two groups in the GEO dataset. **(F)** Time-dependent ROC analysis was conducted to confirm the prediction accuracy of the risk score in the GEO dataset.

To verify that the ATG prognostic model established from TCGA cohort harbored similar prognostic values among different populations, the dataset GSE84437, which contains the clinical information of 433 patients with GC, was utilized as the validation dataset. Patients in the GEO cohort were divided into two risk groups by applying the same median risk score used for TCGA cohort. Both the PCA and t-SNE analysis resulted in a sufficient separation between the groups, which was in line with TCGA cohort findings (**Figures 4A, B**). The distribution of the risk scores and patient survival status was generally consistent with that of the training cohort (**Figures 4C, D**). The K–M curves also showed a difference in survival rates between the groups in the GEO testing cohort (*p*< 0.001, **Figure 4E**). In addition, ROC analysis of the validation cohort suggested the forecasting performance of the established model (**Figure 4F**). Overall, the prognostic model proved to have the favorable discriminative ability. Furthermore, the HPA database was applied to validate the expression of genes used in this model at the protein level. The results revealed that compared with those in normal tissues, SNCG and APOD were expressed at higher levels in GC tissues (**Figure S2-A**). The GEO datasets were employed for additional validation. The results suggested that the expression levels of CYTL1, MATN3, and SERPINE1 were elevated in GC tissues (**Figure S2-B**).

### Assessment of the independent prognostic value of the model

**Figure 5:**
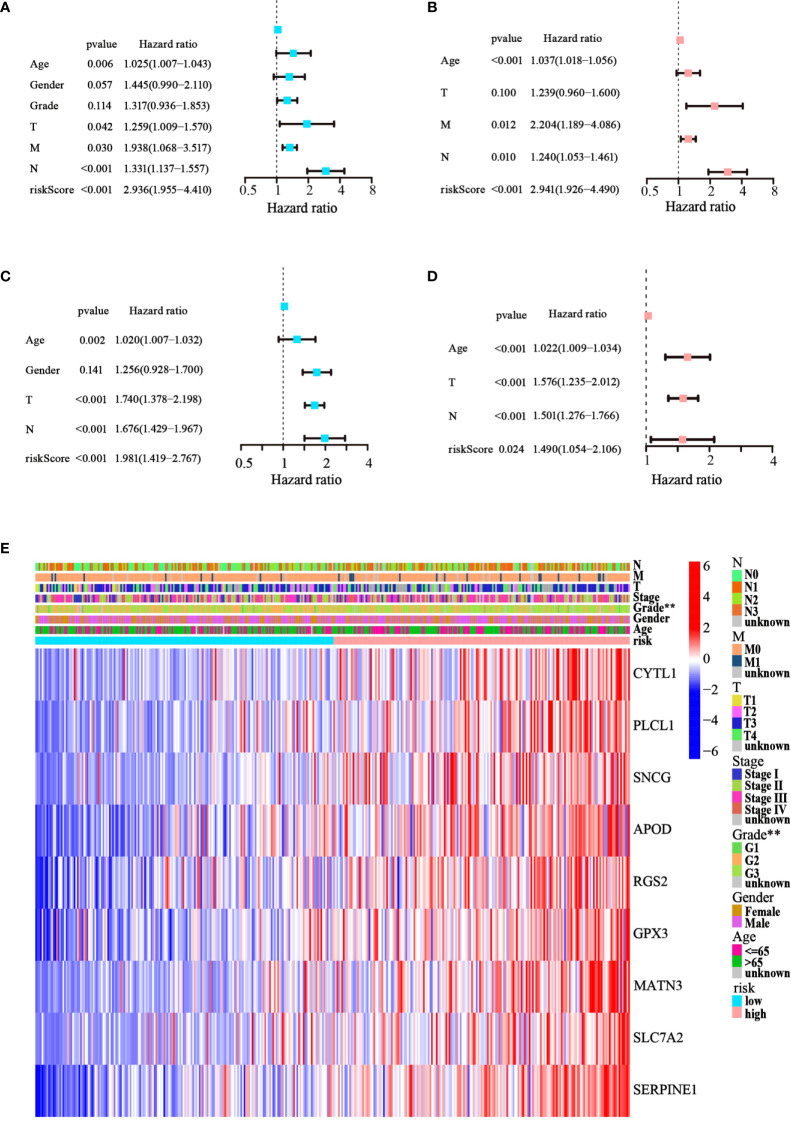
The model’s prognostic significance was determined by Cox regression analysis in GC patients. **(A, B)** Univariate and multivariate Cox regression analysis of risk score and relevant clinical features in TCGA dataset. **(C, D)** Univariate and multivariate Cox regression analysis of risk score and relevant clinical features in the GEO dataset. **(E)** Heatmap of gene expression levels and clinicopathological characteristics variations in the two risk groups. (**p< 0.01).

Cox regression analysis was used to test whether the risk score generated from the model was an independent prognostic factor for GC. The risk score was related to unfavorable outcomes in both the training and testing cohorts, as demonstrated by univariate Cox analysis (**Figures 5A, C**). Moreover, the multivariate Cox analysis implied that the risk score was still a critical forecaster, even when combined with confounding variables (**Figures 5B, D**). Furthermore, we surveyed the relationship between different risk categories and their clinical traits in TCGA cohort, as depicted in the heatmap in **Figure 5E**. We noticed prominent elevations in the levels of all nine ATGs in the high-risk group, and the grade was divided differently between the risk groups (*p*< 0.01). These results demonstrate that the nine-ATG signature can be viewed as an independent prognostic factor for assessing the risk of GC.

### Functional enrichment analysis of the DEGs

**Figure 6:**
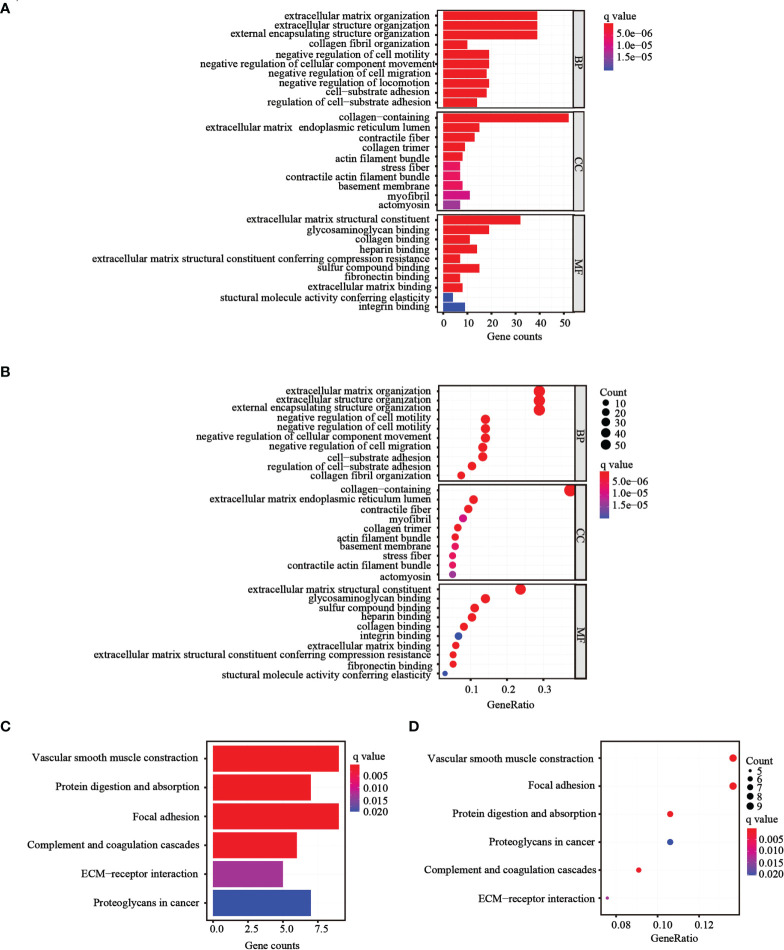
Functional analysis performed in the two risk groups in TCGA cohort. **(A, B)** Bar plot and bubble graph for GO enrichment of DEGs in the two risk groups. **(C, D)** Bar plot and bubble graph for KEGG analysis of DEGs in the two risk groups.

To further determine whether there was variation in gene functions and pathways between the risk groups, 139 differentially expressed genes (DEGs) were identified. GO and KEGG analyses were performed based on the DEGs. Many of these DEGs were found to be involved in biological processes, such as extracellular matrix organization, extracellular structure organization, and external encapsulating structure organization (*p<* 0.05, **Figures 6A, B**). KEGG analysis revealed that the DEGs were mostly involved in vascular smooth muscle contraction and focal adhesion (*p<* 0.05; **Figures 6C, D**).

### Comparison of immune status between subgroups

**Figure 7:**
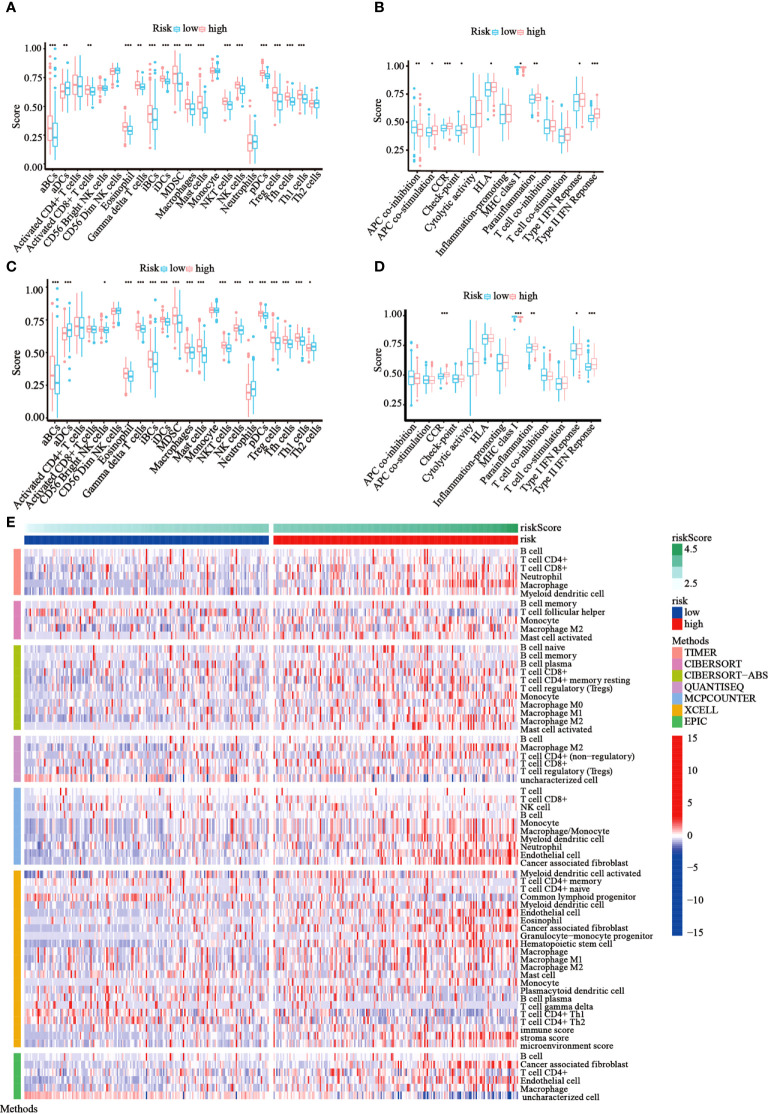
Immune status among the two risk groups was compared in TCGA and GEO cohorts. **(A, B)** The ssGSEA scores of 22 immune cells and 13 immune-associated pathways between the risk groups were exhibited as boxplots in TCGA cohort. **(C, D)** The ssGSEA scores of 22 immune cells and 13 immune-associated pathways between the risk groups were presented as boxplots in the GEO cohort. **(E)** A heatmap of immune cell types enrichment combining all 7 immune score algorithms in TCGA cohort. (*p< 0.05; **p< 0.01; ***p< 0.001).

Following functional analysis, we further explored the correlation between immune infiltration and risk scores. We first assessed the levels of 22 distinct immune cell functions and 13 different immune-related pathways in TCGA cohort using ssGSEA. Compared to those in the low-risk subgroup, we noticed that the high-risk subgroup generally had higher infiltration levels of immune cells, especially activated B cells (aBCs), Eosinophil, immature B cells (iBCs), immature dendritic C cells (iDCs), Myeloid-derived suppressor cells (MDSCs), macrophages, mast cells, natural killer T cells (NKT), natural killer cells (NK), Plasmacytoid dendritic cells (pDCs), regulatory T cells (Tregs), T follicular helper cells (Tfh), and type-I T-helper cells (Th1) (**Figure 7A**). Moreover, the majority of immune-related pathways showed increased activity in the high-risk group, except for APC co-inhibition and MHC class I pathways (**Figure 7B**). Similar outcomes were observed with the GEO cohort (**Figures 7C, D**). Furthermore, we estimated the immune cell populations between the risk groups in TCGA cohort by employing different algorithms, such as TIMER, CIBERSORT, CIBERSORT-Absolute, quanTIseq, MCPcounter, xCell, and EPIC. A heatmap was drawn to show the combined results (**Figure 7E**), which showed a significant difference in the immune infiltration levels between the risk groups. Thus, we conjectured that this might be one of the reasons for the differences in prognoses between high- and low-risk patients.

### Construction and assessment of the nomogram using TCGA cohort

**Figure 8:**
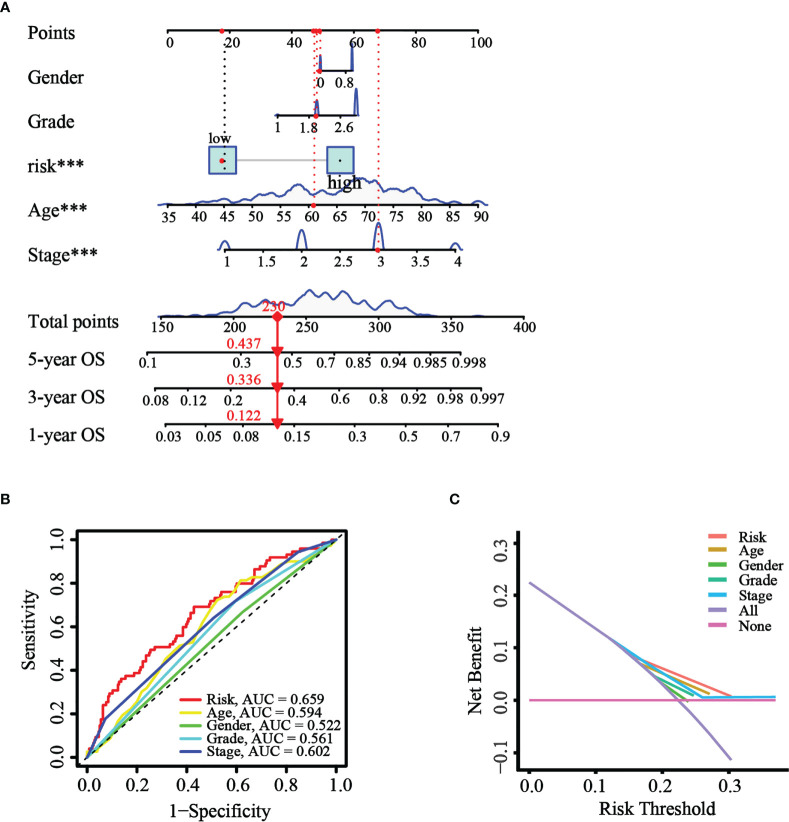
Construction and validation of the nomogram. **(A)** The nomogram was constructed with different factors (risk score, age, gender, grade, and stage). **(B)** The comparison of ROC curves of different factors at 1 year. **(C)** The DCA curve of the significant variables and the nomogram.

Based on the results of univariate and multivariate Cox analysis, a nomogram predicting the overall survival of GC patients was established with TCGA cohort (**Figure 8A**). The risk score was observed to have a significant impact on survival prediction, indicating that the risk model was truly a good predictor of survival. We then compared the AUC of ROC curves of all significant factors (risk score, age, gender, grade, and stage) at 1 year. The results showed that the ROC AUC value for the risk score was higher than those for the other factors (**Figure 8B**), which demonstrated that the risk score of the model had better clinical predictive power. Additionally, the DCA curve revealed that the risk score showed better prognostic capacity than other significant variables (**Figure 8C**). These results indicated the good predictive performance of our model.

### A combination of autophagy inhibitors and DDP inhibits growth and increases ROS in GC cells

**Figure 9:**
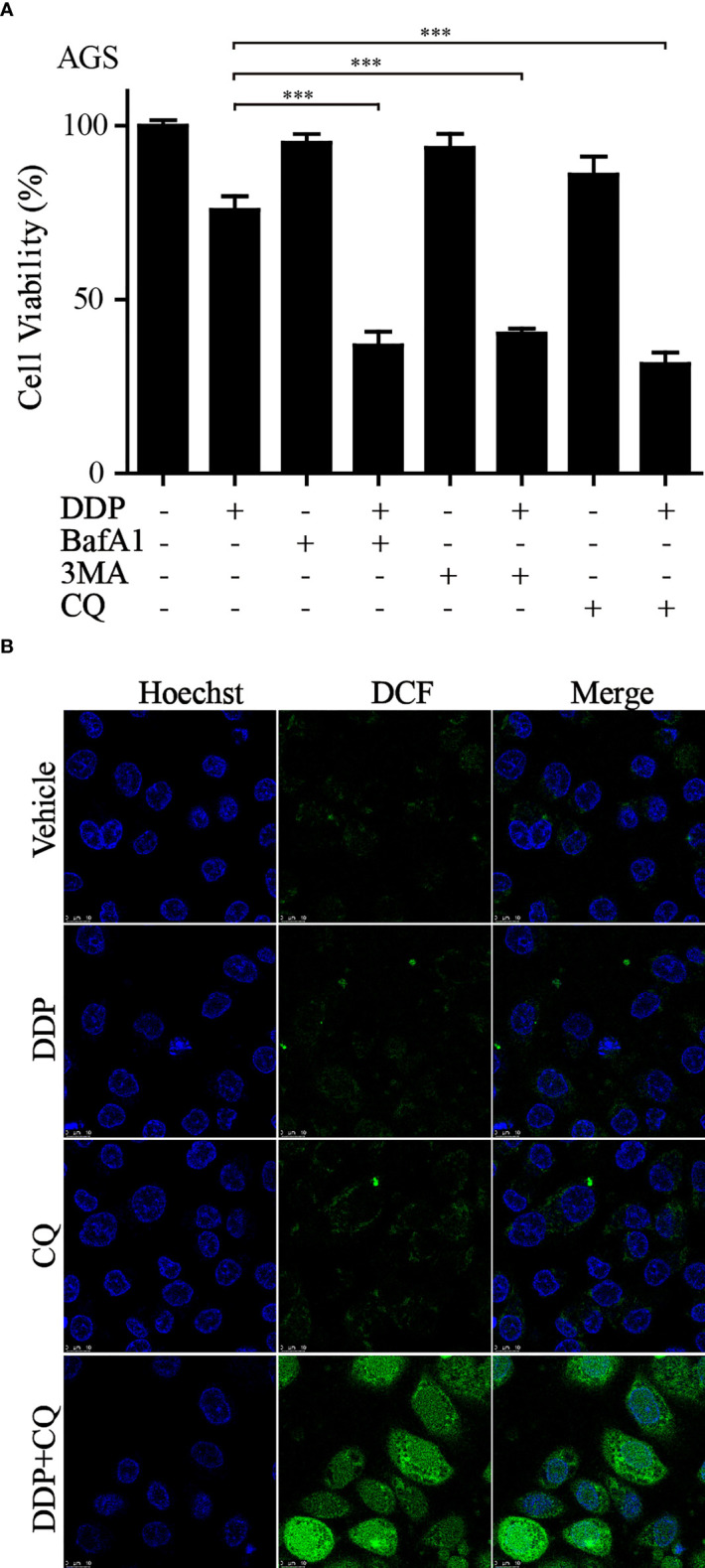
Inhibition of autophagy increased sensitivity to DDP and exacerbated ROS accumulation **(A)** AGS cells were treated with DDP, autophagy inhibitors, or autophagy inhibitors combined with DDP. CCK-8 assay was utilized to examine the cell viability (***p< 0.001). **(B)** ROS production was measured by the DCF-DA probe through confocal imaging with indicated treatment.

It is well known that autophagy has a dual role in the tumor. Our current results suggested that the upregulation of autophagy-related gene expression is closely related to the poor prognosis of GC. Previous studies have shown that the activation of autophagy might lead to chemotherapeutic drug resistance and result in a poor prognosis for GC patients (29). Our earlier work found that the combination of dihydroartemisinin and DDP could effectively improve the sensitivity of tumor cells to DDP and significantly decrease its effective concentrations (30). Therefore, we reasoned that it would be of interest to further investigate whether suppressing autophagy could enhance the cytotoxicity of DDP in GC cells. CCK-8 assays showed that compared to that with DDP or autophagy inhibitors (3MA, BafA1, or CQ) alone, autophagy inhibitors combined with DDP significantly decreased cell viability (**Figure 9A**). We further measured the levels of autophagy-related proteins by western blotting in GC cells administered these treatments. The results revealed that the conversion of LC3, from LC3-I to LC3-II, was significantly increased after applying the combination of DDP and CQ. Further, the accumulation of LC3-II was a consequence of blocking autophagic flux (**Figure S3**). This demonstrated that the autophagic flow was blocked by autophagy inhibitors. These results confirmed that the inhibition of autophagy could restore sensitivity to DDP and improve the prognosis of GC. In addition, it was reported that the suppression of autophagy could lead to ROS accumulation and DNA damage and result in cell death (31). Therefore, cellular ROS levels were detected using the DCF-DA probe. Confocal fluorescence images revealed that compared with those in the DDP and CQ alone groups, ROS levels were significantly increased in the combined treatment group (**Figure 9B**). These results were consistent with our analysis and provided evidence that our model could predict the prognosis of GC patients at the cellular functional level.

## Discussion

Approximately 50% of GC cases occur in China, the vast majority of which are frequently diagnosed at an advanced stage (32). Despite advances in the application of multimodal treatments for GC, the overall survival rate of patients remains far from satisfactory (2). Currently, prevention and personalized treatment are proposed as the optimum solutions to lower GC mortality rates (33). Therefore, the identification of novel and feasible prognostic biomarkers and therapeutic targets could lead to a potential breakthrough in ameliorating the poor outcomes and improving the survival of patients with GC.

Early studies have confirmed that autophagy participates in tumorigenesis and development, and the dysregulation of autophagy might contribute to a variety of diseases, including GC (34–36). Researchers have uncovered a wide expression range for the beclin-1 protein (the master regulator of autophagy) in GC but barely any expression in the normal gastric mucosa (37–39). Autophagy-related gene 5 (*ATG5*), an indispensable constituent of autophagosomes, was found to be overexpressed in GC and associated with an unfavorable clinical outcome owing to its association with chemoresistance (40). Although some ATGs have been investigated in GC, few studies have focused on how holistic ATGs regulate GC progression and prognosis. Accordingly, our work was aimed at exploring the mechanism underlying the effect of ATGs in GC and developing a new molecular-based risk model to improve prognostication for GC.

We first identified differentially expressed ATGs from GC samples in TCGA cohort. Consensus clustering analysis was performed to classify samples into two clusters. We initially screened the DEGs associated with clinical features, including prognosis. To further evaluate the prognostic value of these ATGs, we combined univariate Cox regression analysis and LASSO regression analysis to establish a prognostic risk model for risk and outcome prediction. Among the nine genes, we found that all of them were risk factors for GC. Cytokine-like 1 (*CYTL1*) has been identified as a new type of chemotactic cytokine involved in regulating tumorigenesis (41–43). Moreover, CYTL1 might serve as a tumor suppressor with broad inhibitory effects on tumor metastasis and the phosphorylation of STAT3 in multiple tumor models (44). Phospholipase C‐like 1 (*PLCL1*), which is homologous to the PLC family, is associated with tumor growth suppression (45) and metastasis (46). The downregulation of *PLCL1* expression in clear cell renal carcinoma and neuroblastoma was found to be predictive of poor prognosis (47, 48). The expression of gamma-synuclein (*SNCG*), a member of the synuclein family, was found to be upregulated in GC (49) and was also thought to enhance the growth of cervical cancer by activating the AKT pathway (50). Known as an inhibitor of G-protein signaling, the dysregulation of *RGS2* has been implicated in tumor initiation and progression in breast cancer (51), acute myeloid leukemia (52), and prostate cancer (53). In a recent study, it was shown that GPX3 not only suppresses stomach cancer tumor growth but also prevents metastasis (54). Particularly in GC, GPX3 can block the NFкB/Wnt/JNK signaling pathway, thereby suppressing cell migration and invasion (55). Elevated *SERPINE1* expression in GC has been associated with poor outcomes (56). Collectively, these findings are evidence that the nine-gene signature is an indicator of poor prognosis in various cancer types, especially GC; thus, a model based on this signature could improve, to some extent, the prediction of disease risk and outcomes in GC.

We subsequently corroborated the prognostic accuracy and specificity of our model using both the testing and training cohorts, and the results were visualized using K–M and ROC curves. The risk score derived from the model was determined to be an independent prognostic factor for patients with GC. As a key part of the tumor microenvironment, the extracellular matrix (ECM) is reported to be associated with drug resistance and immune suppression (57, 58) and can form a physical barrier to reduce infiltration (59). Previous studies have demonstrated that ECM degradation could facilitate the migration and dissemination of malignant cells (60, 61). It is reasonable to speculate that genes comprising the established model could influence the composition of the tumor microenvironment. Accordingly, we compared immune infiltration between the low- and high-risk groups. The results revealed that patients in the high-risk group were associated with an immune-activated state. Studies have shown that Treg cells and infiltrating B cells are associated with decreased survival rates and unfavorable prognosis in patients with GC (62, 63). Consistent with this funding, our study showed the obvious enrichment of Treg cells and B cells in the high-risk group. The tumor immune microenvironment is indicative of the immune status of the patient, which probably explains the survival differences between the risk groups.

Although DDP and other platinum drugs have been proven to dramatically improve the prognosis of GC, drug resistance remains a great challenge for GC treatment (3). Previous studies have reported that DDP induces cellular protective autophagy and that inhibiting autophagy could improve DDP chemotherapy (64). It has been shown that autophagy inhibitors can reverse chemotherapy resistance in GC (65). Our studies found that the combination of autophagy inhibitors and DDP could markedly inhibit cell viability and induce ROS production. These results will provide a fresh idea for solving GC drug resistance.

In summary, we have developed a reliable prognostic model based on ATGs in GC. Even though the risk model exhibited remarkable predictive power, it needs to be optimized. Owing to the retrospective nature of the present study, some degree of bias was likely introduced. Further, the potential interactions among these model genes should be explored *via in vitro* and *in vivo* experiments to better understand the exact molecular mechanisms underlying their effects on the GC process. Above all, this study represents the application of consensus clustering to stratify patients with GC based on the expression profile of ATGs, which was found to be relevant to the tumor immune microenvironment. Moreover, our work might help to understand how autophagy affects the prognosis of GC and is expected to help clinicians develop new treatment strategies.

## Data availability statement

The original contributions presented in the study are included in the article/**Supplementary Material**. Further inquiries can be directed to the corresponding authors.

## Author contributions

JD, YW, and YL designed this work and revised this manuscript. JX and PZ designed *in vitro* experiments, LY, LTa, SC, and LTo prepared the data. HX, BX, and JH integrated and analyzed the data. HX, BX, and JH wrote this manuscript. All authors contributed to the article and approved the submitted version.

## Funding

This research was supported by Zhejiang Public Welfare Technology Application Research Project (Grant Nos. LGF22H160027, LGF21H010008, LGF20H080005).

## Conflict of interest

The authors declare that the research was conducted in the absence of any commercial or financial relationships that could be construed as a potential conflict of interest.

## Publisher’s note

All claims expressed in this article are solely those of the authors and do not necessarily represent those of their affiliated organizations, or those of the publisher, the editors and the reviewers. Any product that may be evaluated in this article, or claim that may be made by its manufacturer, is not guaranteed or endorsed by the publisher.

## Supplementary material

Supplementary Table 1 232 autophagy-related genes.

Supplementary Table 2 148 differentially expressed genes.

Supplementary Figure 1 Heatmaps of Consensus clustering results for different k-values (k= 3 to 9).

Supplementary Figure 2 The modeling genes showed a higher expression level in GC tissues compared with normal tissues. (A) Representative images of immunohistochemical (IHC) staining of SNCG and APOD in GC and normal tissues (Human Protein Atlas). (B) The GEO data analysis revealed that the expression levels of CYTL1, MATN3, and SERPINE1 were elevated in GC tissues. (**p*< 0.05; ***p*< 0.01; ****p*< 0.001).

Supplementary Figure 3 AGS cells were treated with DDP, CQ, or the combination of DDP and CQ for 5 h, and the expression level of LC3 was detected by western blot.
