# Supplementary figures and images for: Development of a novel autophagy-related gene model for gastric cancer prognostic prediction
Source: Front Oncol. 2022 Oct 7;12:1006278. doi: 10.3389/fonc.2022.1006278 (PMC9585256; doi:10.3389/fonc.2022.1006278)

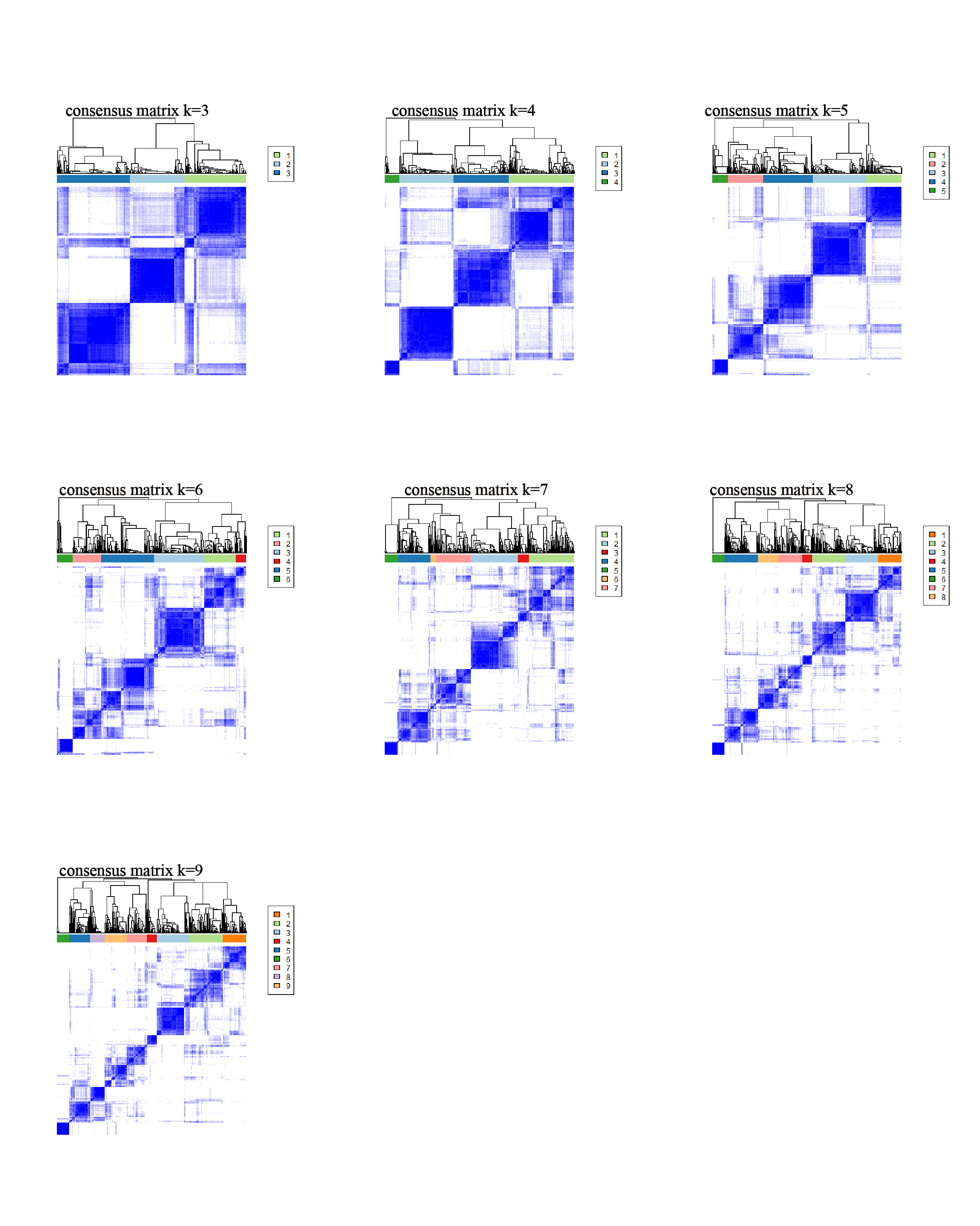

Supplement: Supplementary Figure 1 — Heatmaps of Consensus clustering results for different k-values (k= 3 to 9). [file Image_1.jpeg]

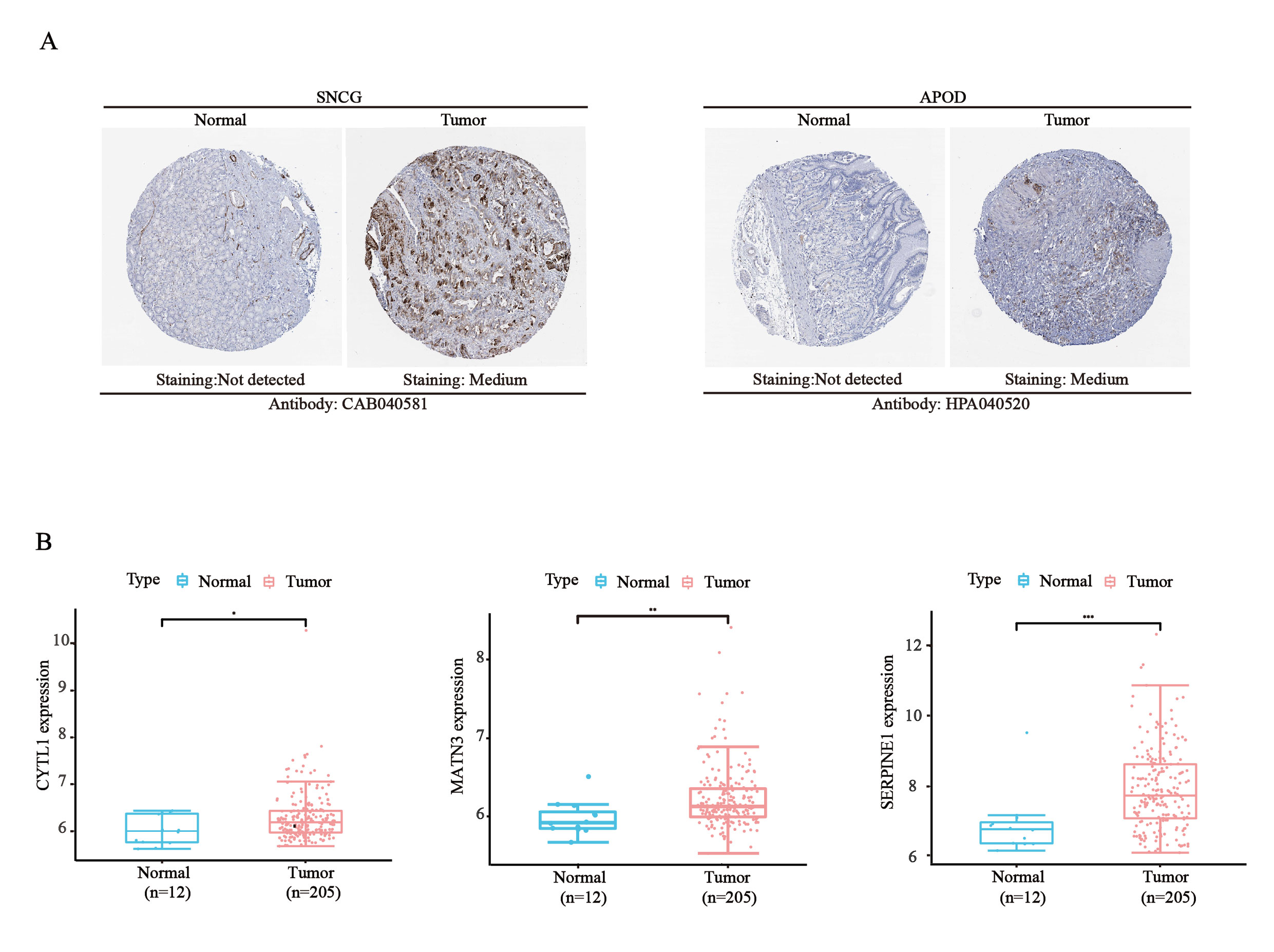

Supplement: Supplementary Figure 2 — The modeling genes showed a higher expression level in GC tissues compared with normal tissues. (A) Representative images of immunohistochemical (IHC) staining of SNCG and APOD in GC and normal tissues (Human Protein Atlas). (B) The GEO data analysis revealed that the expression levels of CYTL1, MATN3, and SERPINE1 were elevated in GC tissues. (*p< 0.05; **p< 0.01; ***p< 0.001). [file Image_2.jpeg]

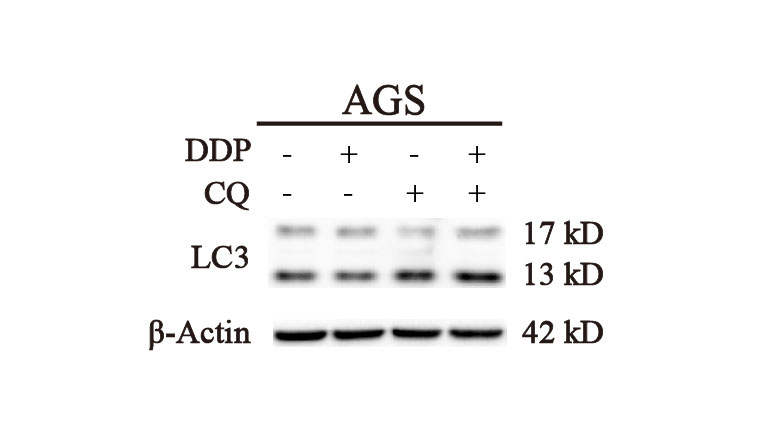

Supplement: Supplementary Figure 3 — AGS cells were treated with DDP, CQ, or the combination of DDP and CQ for 5 h, and the expression level of LC3 was detected by western blot. [file Image_3.jpeg]
